# Unraveling the Link Between Borderline Personality Disorder and Attention Deficit Hyperactivity Disorder

**DOI:** 10.1192/j.eurpsy.2025.293

**Published:** 2025-08-26

**Authors:** V. A. Barata, P. Gonçalves

**Affiliations:** 1 Hospital Professor Doutor Fernando Fonseca, Amadora, Portugal

## Abstract

**Introduction:**

Attention Deficit Hyperactivity Disorder (ADHD) and Borderline Personality Disorder (BPD) are common psychiatric conditions, with comorbidity rates ranging from 18% to 34% in adults. Both disorders share symptoms such as impulsivity, emotional dysregulation, and interpersonal dysfunction, complicating differential diagnosis. Their frequent co-occurrence may suggest overlapping developmental and environmental risk factors as well as common underlying mechanisms. Particularly for BPD patients, recognizing the shared characteristics between ADHD and BPD may help reduce the stigma surrounding this disorder and eventually lead to a broader range of pharmacological treatment options.

**Objectives:**

This review aims to explore the links between ADHD and BPD, focusing on their symptomatology overlap, comorbid presentation, shared risk factors and treatment insights in adults.

**Methods:**

A narrative literature review was conducted using the keywords “ADHD”, “borderline personality disorder”, “comorbidity” and “adults” in PubMed and Google Scholar databases.

**Results:**

Findings reveal that ADHD and BPD share several risk factors, including genetic predispositions and early-life adversities. Early-life adversity, particularly trauma, is a shared risk factor; however, the type and timing of adversity seem to play differential roles in developing ADHD and BPD symptoms. In terms of clinical presentation, ADHD is characterized by more severe impulsivity compared to non-comorbid BPD, whereas BPD features more severe difficulties in emotional regulation compared to non-comorbid ADHD. The comorbid ADHD+BPD presentation is marked by heightened severity in both impulsivity and emotional instability. The traditional view of ADHD as an early-onset disorder and BPD as a late-onset personality disorder is increasingly questioned, prompting calls for a dimensional diagnostic approach. Recent studies highlight the potential of ADHD medication, particularly stimulant compounds, in reducing suicidal behavior among BPD patients exhibiting ADHD symptoms. Such medications have been found to lower the risk of suicidal behavior in these patients, while other treatments (e.g., antidepressants, antipsychotics, mood stabilizers) did not show similar protective effects.

**Conclusions:**

This review highlights the complex interplay between ADHD and BPD, emphasizing that both disorders should be considered within a dimensional framework rather than as separate categorical entities. Shared symptoms and risk factors underscore the need for integrated treatment approaches that address the combined symptomatology of ADHD and BPD. Future research should focus on understanding the developmental trajectories of these disorders, refining diagnostic criteria, and evaluating the effectiveness of combined treatment strategies.

**Disclosure of Interest:**

None Declared

